# Effects of Easy-to-Use Protein-Rich Energy Bar on Energy Balance, Physical Activity and Performance during 8 Days of Sustained Physical Exertion

**DOI:** 10.1371/journal.pone.0047771

**Published:** 2012-10-18

**Authors:** Minna M. Tanskanen, Klaas R. Westerterp, Arja L. Uusitalo, Mustafa Atalay, Keijo Häkkinen, Hannu O. Kinnunen, Heikki Kyröläinen

**Affiliations:** 1 Department of Biology of Physical Activity, University of Jyväskylä, Jyväskylä, Finland; 2 Department of Human Biology, Maastricht University, Maastricht, The Netherlands; 3 Department of Clinical Physiology and Nuclear Medicine, Helsinki University Hospital, Helsinki, Finland; 4 Institute of Biomedicine, Physiology, University of Eastern Finland, Kuopio, Finland; 5 Polar Electro Oy, Kempele, Finland; University of Valencia, Spain

## Abstract

**Background:**

Previous military studies have shown an energy deficit during a strenuous field training course (TC). This study aimed to determine the effects of energy bar supplementation on energy balance, physical activity (PA), physical performance and well-being and to evaluate ad libitum fluid intake during wintertime 8-day strenuous TC.

**Methods:**

Twenty-six men (age 20±1 yr.) were randomly divided into two groups: The control group (n = 12) had traditional field rations and the experimental (Ebar) group (n = 14) field rations plus energy bars of 4.1 MJ•day^−1^. Energy (EI) and water intake was recorded. Fat-free mass and water loss were measured with deuterium dilution and elimination, respectively. The energy expenditure was calculated using the intake/balance method and energy availability as (EI/estimated basal metabolic rate). PA was monitored using an accelerometer. Physical performance was measured and questionnaires of upper respiratory tract infections (URTI), hunger and mood state were recorded before, during and after TC.

**Results:**

Ebar had a higher EI and energy availability than the controls. However, decreases in body mass and fat mass were similar in both groups representing an energy deficit. No differences were observed between the groups in PA, water balance, URTI symptoms and changes in physical performance and fat-free mass. Ebar felt less hunger after TC than the controls and they had improved positive mood state during the latter part of TC while controls did not. Water deficit associated to higher PA. Furthermore, URTI symptoms and negative mood state associated negatively with energy availability and PA.

**Conclusion:**

An easy-to-use protein-rich energy bars did not prevent energy deficit nor influence PA during an 8-day TC. The high content of protein in the bars might have induced satiation decreasing energy intake from field rations. PA and energy intake seems to be primarily affected by other factors than energy supplementation such as mood state.

## Introduction

Ready-to-eat meal rations are commonly used during the military field training [1]. However, previous military studies have shown an energy deficit during a strenuous field training course [2], [3], which induces loss of body mass and fat-free mass. In many military studies energy restriction is caused by a purpose as an additional stressor [3]–[6], but also underconsumption is a common problem [1], [7], [8]. Even though unfamiliar stressful situations might decrease energy intake [9], environmental factors has an important role in determining food intake and choice [10] and food acceptability has been found to have an impact on food intake in the field [10]. Moreover, multiple stressors of sleep and caloric deprivation with physical and psychological stress have deteriorated effect on mood state [5].

Energy intake (EI) itself has been found to have an influence on voluntary physical activity, increasing with high-energy intake [11], [12]. Furthermore, energy balance is an important factor in sustaining training load and maintaining high performance during strenuous military training [13]–[15]. Demanding training is risk factor for upper respiratory tract infection (URTI) among athletes [16] and soldiers [17], and energy deficit has been hypothesized to be one reason for URTI [18], however, scientific evidence is lacking. In a study by Costa et al. [19] consumption of a high carbohydrate diet throughout a strenuous training period had a favorable effect on markers of immune activity and, thereby, reduced susceptibility to URTI. Similarly in another study by Flakoll et al. [20], post exercise protein supplementation resulted in reduced bacterial/viral infections during military basic training period. In both of these studies, the supplementation group also received higher energy intake. Therefore, it cannot be concluded whether this phenomenon occurs due to increased protein or carbohydrate intake or just a result of increased energy intake. On the other hand, high-protein diets have been found to attenuate a decrease in fat-free mass (FFM) during negative energy balance induced by a decrease in energy intake among obese individuals [21] and also among athletes [22]. However, high-protein intake has not been found to attenuate a decrease in FFM when energy deficit is induced by high energy expenditure [23].

There are few published energy supplements studies during military field training in purpose to assure energy balance [12], [14], [15], [24]–[26], however, none of this have used protein-rich supplement. Thus the aim of this study was to determine the effects of an additional easy-to-use protein-rich energy bar supplement of 4.1 MJ·d^−1^ on energy balance, physical activity, body composition, physical performance and subjective experiences of hunger, URTI, mood state and overall well-being during the 8-day military training course (TC) in a cold environment. In the present study, the aim was not to cause energy restriction and neither force the conscripts to eat the all provided food. Instead of that the aim was to observe the effect of military training in terms of self-selected diet. An additional purpose was to define whether an ad libitum fluid intake is sufficient during winter training. We hypothesized that energy supplement of 4.1 MJ·d^−1^ may increase the total energy intake, improve ability to maintain physical training load and sustain FFM during TC. Furthermore, we hypothesized that an energy supplement will sustain physical performance, enhance mood state and overall well-being and decrease symptoms of URTI.

## Methods

### Ethics Statement

All participants were fully informed of the experimental protocol and gave their written consent to participate in the study. They were also advised of their right to withdraw from the investigation at any time. The study protocol was approved by the Finnish Defence Forces, and the Ethical Committees of the University of Jyväskylä and the Kainuu region of Finland.

#### Participants

Twenty-six men (aged 20±1 yr.) volunteered to participate in the present study during their 8-day field training course (TC). The participants were randomly divided into two groups: the control group (n = 12) were allowed to eat only traditional field rations and the experimental (Ebar) group (n = 14) field rations plus energy bars of 4.1 MJ•day^−1^. The two groups did not differ according to the baseline characteristics (Table 1).

**Table 1:** Physical characteristics of the control and Ebar groups before the training course.

	Control (N = 12)	Ebar (N = 14)
Age (years)	20.2±1.6	19.6±0.5
Height (cm)	177.8±6.6	178.9±7.9
Body mass (kg)	71.3±7.4	71.2±7.4
Fat mass (kg)	12.1±3.8	10.4±1.9
Fat-free mass (kg)	70.5±7.4	70.5±7.3
Fat%	16.8±4.5	14.6±2.4
BMI (kg·m^−2^)	22.6±2.1	22.2±1.1
TBW (L)	43.4±4.5	44.6±4.7
12-minute running test (m)	2814±208	2930±178
VO_2_max (mL·kg^−1^·min^−1^)^aa^	52±5	54±1

Values are mean ± SD.

aa Estimated from 12-minute running test: VO_2_max (ml·kg^−1^·min^−1^)  =  running distance (m) - 504.9/44.7 [57].

### Experimental Design

The study took place during the winter in Finland, when daily outdoor temperatures ranged from −16°C to −1°C, with an average of −4°C (data from the local weather station). During the study, the participants completed 8-days field training, which consisted of a variety of military-relevant tasks and skills training, sustained aerobic exercise from skiing and patrolling activities. During TC, participants carried combat gear weighing 60–70 kg including their clothes and food rations. The participants were randomly divided into two groups: a control group (N  = 12) and an energy supplement group (Ebar, N  = 14) of which the latter received an additional energy supplement of 4.1 MJ•day^−1^ during the 8-day TC. Water intake was ad-libitum. However, participants were not permitted to use any other extra nutritional supplements throughout the study. Physical performance was studied before [six days before (-6d PRE); one day before (PRE)], during (MID) and after [one day after, POST; three days after (+3d POST)] TC. The measurements included a 3 km combat loaded field running, vertical jump, anaerobic power and handgrip strength. The participants have already done intensive military training for six months and were familiar with the stressors during TC. In addition, they were familiarized with the performance tests, and the jump and hand-grip strength tests were performed twice before (-6 PRE and PRE) the training course for minimizing the changes due to learning. In addition, mood state, body composition and physical activity were studied together with energy and water intake recordings. The experimental protocol is presented in Table 2.

**Table 2:** The experimental protocol.

			Intensive training course									
Day	-6 PRE	PRE	1	2	3	4	5	6	7	8	POST	+3 POST
Test												
Body mass	X	X					X				X	X
Fat-free mass		X										
Fat mass		X									X	
Questionnaires	X	X					X				X	X
Vertical jumpAnaerobic powerHand-grip test	X	X					X				X	
	X	X					X				X	
	X	X					X				X	
3 km running test		X									X	
Physical activity			X	X	X	X	X	X	X	X		
Energy and water intake			X	X	X	X	X	X	X	X		
Energy bar supplement			X	X	X	X	X	X	X	X		
Deuterium dose		X								X		
Urine samples		X	X							X	X	

### Anthropometry and Body Composition

Body mass (BM) was measured with an accuracy of 0.01 kg (Inbody720 body composition analyzer, Biospace Co. Ltd, Seoul, Korea). The measurements were performed between 6∶00 and 7∶00 a.m. after an overnight fast and after voiding with no exercise for 12 hours prior to the test commencing, except the measurements in MID of TC. The participants were barefoot and they wore T-shirts and shorts. The BMmean (an individual average of PRE, MID and POST) was used for calculation energy intake variables per BM. Body height was measured to the nearest 0.5 cm using a wall-mounted stadiometer. Fat-free mass (FFM) was calculated from total body water (TBW) as follows: FFM  =  TBW/0.732 [27]. Fat mass (FM) was calculated as BM – FFM, and percentage of body fat as FM/BM x 100. TBW was measured with deuterium dilution according to the Maastricht protocol [28]. Briefly, at 10∶00 p.m., before the measurements and after collecting a baseline urine sample, the participants drank a weighed mixture of ^2^H_2_O. Participants consumed no foods or fluids for 10 hours after dose administration, during the overnight equilibration of the isotope with the body water. Subsequent urine samples were collected from the second and third voiding in the morning of day 1. On study days 9 and 10 the process was repeated. TBW was calculated as the ^2^H dilution space divided by 1.04, correcting for exchange of the ^2^H label with nonaqueous H^+^ of body solids [29] **.**

### Energy and Water Intake and Water Loss

The average daily energy from a field ration was 17.9 MJ·d^−1^ with the average macronutrient composition in energy of 14 E% protein, 58 E% carbohydrate and 28 E% fat. The participants in the Ebar group were advised to eat five commercial energy bars per day totaling 4.1 MJ·d^−1^ extra energy. The energy bars weighed 55 g/bar with the average macronutrient composition in energy of 32 E% protein, 46 E% carbohydrate and 22 E% fat. The total provided energy for the Ebar group was 22.0 MJ·d^−1^ (17 E% protein, 56 E% carbohydrate and 27 E% fat). For studying energy intake (EI) and water intake, the participants kept daily pre-filled ration diaries. The pre-filled food diary included information about the ration served, and thus the participants were only required to record the amount of food and fluid that they consumed. The participants had completed these diaries before the study to ensure that they were familiar with reporting food and water intake. In addition, they were advised to be as accurate as possible in recording the amount food and fluid consumed. Furthermore, all the food wraps and rations that were not consumed were collected. Daily nutritional consumption was quantified from manufacturer-supplied ingredient labels and by the computer programme Nutrica®software (version 3.11, Finland) using the information from ration diaries, wraps and returned rations.

*Total water intake* was calculated from reported food and water intakes and metabolic water as follows: Total water intake  =  fluid intake + water content of food + metabolic water. The amount of metabolic water was estimated from protein, fat and carbohydrate intake and from the change in BM. Oxidation water is 0.41 mL•g^−1^ for protein, 1.07 mL•g^−1^ for fat and 0.6 mL•g^−1^ for carbohydrate [30]. A change in BM of 1 kg was assumed to be a change of 0.75 kg FM and 0.25 kg FFM. FM was assumed to be pure fat and FFM 73% water and 27% protein [31].

*Water loss* over TC was measured using the deuterium elimination method [30]. Deuterium elimination was calculated from two urine samples after dosing (at day 1 in the morning) and two samples at the end of the training period (day 8 in the evening). Water balance was calculated as follows: Water balance  =  total water intake – water loss.

### Energy Expenditure, Energy Balance and Energy Availability

Total energy expenditure (EE) was calculated by a method of intake/balance using EI and changes in body energy stores (ΔES); EE  =  EI – ΔES [8]. ΔES were calculated from changes in FFM and FM between PRE and POST values. Energy equivalents used for protein and fat were 18.4 and 39.8 kJ·g^−1^, respectively [31].

Energy balance was calculated as follows: Energy balance  =  EI – EE. To differentiate the need of energy intake for subjects with a different body mass, energy availability was calculated as follows: EI/BMR, where BMR  =  basal metabolic rate estimated by the equation of Schofield et al. [32]. Energy availability describes the fraction of energy that is left for physical activity, while BMR has been taken into account.

### Physical Activity

Physical activity (PA) was measured using a customized version of the Polar AW200 Activity monitor that was worn on the non-dominant wrist. AW200 has been found useful and accurate for the measurement of EE during long term exercise [33]. AW200 contains a uniaxial accelerometer, of which the signal is bandpass filtered (0.3–3.0 Hz) reducing sensitivity to repeated low-intensity hand movements. The device counts hand movements if their acceleration exceeds 1.0 m·s^−2^ [34]. Epoch length was set at one minute and a curvilinear equation was used to transform activity counts to metabolic equivalents (1–16 MET), which were further adjusted by body height. Among the participants in the military environment, weekly energy expenditure estimated with AW200 correlated well (r  = 0.78) to that obtained with doubly labeled water (unpublished data, see [13]. Periods that contained no single movement during 30 min were classified as non-wear time and excluded from the recordings. PA by each minute was classified into either (1) rest ≤1.0 MET, (2) sitting  = 1–2 MET, (3) standing  = 2–3.5 MET, moderate activity  = 3.5–6 MET, (4) vigorous activity ≥6 MET or (5) active time ≥3.5 MET ( =  moderate + vigorous activity). REST (i.e. sleep) was selected, if accelerometer showed no hand movements within more than 50% of a 10- min moving window. Participants were only included for analysis, if their activity recordings covered more than 90% of the entire recording time. The activity devices were collected on day 5 for data download, and redistributed within 5∶30 h:min. Data was pooled into three periods: days of 1–5, days of 5–8 and total time of the TC days of 1–8. Data recordings lasted from 9∶00 a.m. on day 1 to 20∶00 p.m. on day 8.

### Physical Performance

The vertical jump test, anaerobic power test and handgrip strength were performed -6d PRE, PRE, MID and POST the TC. Prior to these tests each participant was required to complete a standardized warm-up of 5-min. After warm-up, the participant performed either hand-grip or jump test with 5 min rest between the tests. The follow-up tests for each participant were always performed in the same order and at the same time of day. A standardized warm-up of 1 km walking for 3-km running test started immediately after completion the hand-grip and jump tests in PRE and POST having consumed a similar diet before each testing session.

*Leg explosive power* was assessed with a static vertical jump test using a contact mat (Newtest Powertimer, Newtest Oy, Oulu, Finland). Jump test has been found a sensitive indicator for detecting decrements in physical performance after short [35] and long period of military field training [3]. The participant stood on the contact mat with hands on the hips and the knees flexed at an angle of 90 degrees. Instructions were given for jumps to be performed vertically with the maximal effort, and to land on the mat with knees straight. Hands were kept on the hips during the jump. The participants performed three static jumps with 5-s rest between each jump. The mean flight time of the two best jumps were recorded and the rising height of the center of gravity was assessed. After two min rest, anaerobic power was assessed during a 15-s all-out counter movement jump test on the contact mat. Power was calculated as follows:

Power (W·kg^−1^)  =  ((g^2^) x (T_f_ x 15))/(4n x ((15- T_f_)), where g  =  acceleration of the gravity (9.81 m·s^−2^), T_f_  =  total flight time, n  =  number of the jumps performed in 15 s [36].

*The maximum isometric strength of the hand and forearm muscles* was assessed with the static *hand-grip test* using a dynamometer (SAEHAN, Saehan Corp., Masa, Korea). Three maximum isometric efforts with one minute rest was performed and maintained for about 5 s with the right and left hand with shoulder adducted and neutrally rotated, elbow flexed to 90 degrees and forearm in a neutral position. No other body movement was allowed. The handle of the dynamometer was individually adjusted for each participant. The participants were strongly encouraged to perform with maximal effort. The hand-grip results are presented as the mean of the right and left hand maximum.

*The 3-km running test* with maximal effort was performed on an outdoor track PRE and POST TC while carrying a 20 kg backpack. All participants were instructed to complete the test in the shortest possible time. Heart rate (HR) was recorded continuously using hear rate monitors (RS 800, Polar Electro Oy, Kempele, Finland). Blood lactate was determined before and 1 minute after completion of the exercise from a fingertip blood sample using a lactate analyzer (LactatePro®, Arkray, Japan).

### Questionnaires

The participants rated how they experienced their feelings utilizing five different aspects: 1) hunger on an eight-point Likert scale, 2) physical performance on a seven-point Likert scale and 3) positive and negative mood states on a four-point Likert scale, which included six positive and nine negative items, and the means of both items were calculated [37]. 4) Somatic symptoms were analyzed on a five-point Likert scale; 1 =  not at all, 2 =  one day, 3 = 2–3 days, 4 = 4–5 days, 5 = 6–7 days. The symptoms were subjective ratings of well-being; digestive disorders and reduced appetite, musculo-skeletal, physical complaints and sleep disturbances [38]. The degree of symptoms was determined as the sum of their scores. 5) URTI symptoms were evaluated by the Wisconsin Upper Respiratory Symptom Survey (WURSS-21) [39].

### Statistical Analyses

All data is presented as mean ± SD. The level of statistical significance was set at p<0.05. If assumptions for normality were not met, data were log-transformed before statistical analysis. The untransformed values are shown in the text, tables and figures for giving more meaningful values. Responses to TC, and differences between and within the controls and Ebar participants, were assessed using the mixed-design factorial ANOVA [group (control vs. Ebar) x TC (PRE, MID, POST). Bonferroni as the *Post hoc* analysis was used to identify any significant differences. In addition, the effect of group and TC interaction was calculated, and 95% confidence intervals (CI) were determined. If 95% confidence interval included zero, it was concluded that there was no significant effect. Pearson product–moment correlations were used to observe associations between variables. All statistical analyses were performed with PASW Statistics software (Version 18.0.0. 2009; SPSS Inc., Chicago, IL).

## Results

### Energy Intake and Energy Availability

**Figure 1:**
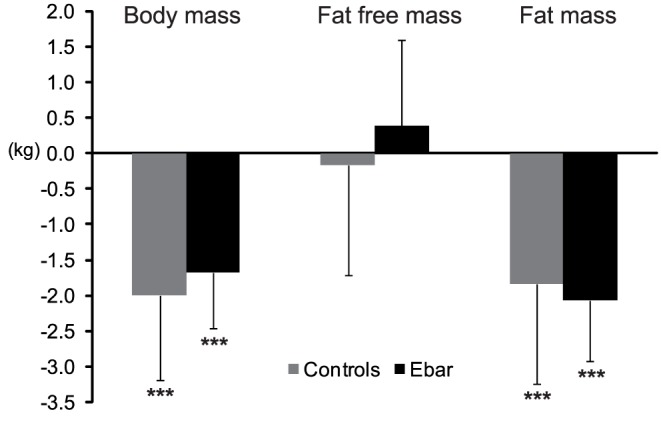
Changes in body composition during the 8-day training course in the control and Ebar groups. *** P<0.001, significant change during the training course.

The Ebar group consumed 58±15% and the controls 57±10% of the provided energy during TC. The estimated energy intake from the field ration did not differ between the groups (Ebar 10.0±1.0 MJ·d^−1^, P = 0.23 vs. control 10.9±1.7 MJ·d^−1^). However, the estimated total energy intake and energy availability were higher (P  = 0.038 and P  = 0.028, respectively) in Ebar compared to the controls (Table 3). Intake of protein was also higher among Ebar (P<0.001) than in the controls (Table 3). Energy balance was negative among all participants and energy balance (Ebar −10.1±3.7 MJ·d^−1^, −43± −12%, P  = 0.056; control −9.2±6.4 MJ·d^−1^, −40± −25%) and estimated energy expenditure (Ebar 23.3±3.2 MJ·d^−1^, P  = 0.060; control 20.2±5.7 MJ·d^−1^) did not differ between the groups. BM and FM decreased in both groups (−2.1% and −16.5%, respectively, P<0.001) during TC while FFM (Figure 1) and TBW remained the same (Ebar 44.9±4.7 L; control 43.4±4.7 L). The estimated fluid intake (control 2.3±0.5 L·d^−1^; Ebar 2.1±0.6 Ld^−1^) and total water intake were similar in both groups, and there was no significant water deficit (Table 3).

**Table 3:** Energy intake, macronutrient composition of the consumed food and water balance during the 8-day training course among the control and Ebar groups.

	Control (N = 12)	Ebar (N = 14)	P
Total energy intake (MJ·d^−1^) a	10.9±1.7	13.3±3.0	**0.025**
Total energy intake (MJ·kg^−1^·d^−1^)	0.16±0.30	0.19±0.43	**0.038**
Energy availability b	1.5±0.3	1.8±0.4	**0.028**
Carbohydrate (g·d^−1^)	359±46	413±84	0.063
Fat (g·d^−1^)	86±21	99±23	0.165
Protein (g·d^−1^)	92±20	149±46	**<0.001**
Carbohydrate (g·kg^−1^·d^−1^)	5.2±1.0	5.9±1.2	0.108
Fat (g·kg^−1^·d^−1^)	1.2±0.3	1.4±0.3	0.196
Protein (g·kg^−1^·d^−1^)	1.3±0.3	2.1±0.6	**<0.001**
Fluid intake (L·d^−1^) c	2.3±0.5	2.1±0.6	0.376
Total water intake (L·d^−1^) d	3.4±0.6	3.3±0.5	0.833
Water loss (L·d^−1^)	−3.5±0.5	−3.3±0.5	0.382
Water deficit (L·d^−1^)	−0.1±0.7	0.0±0.7	0.584

Values are mean ± SD.

a Estimated from food recods.

b Energy availability  =  energy intake/basal metabolic rate estimated by the equation of Schofield et al. [32].

c Estimated from fluid intake records.

d Total water intake  =  fluid intake + water content of food + metabolic water.

### Physical Activity

Physical activity did not differ between the groups during TC. The mean rest during TC was 4∶05±0∶32 h:min per day (range from 3∶00 to 6∶48 h:min), sitting 5∶05±0∶46 h:min (3∶32–6∶53 h:min), standing 9∶10±0∶44 h:min (7∶54–10∶31 h:min), moderate activity 5∶32±1∶09 h:min (3∶46–7∶34 h:min), vigorous activity 0∶06±0∶03 h:min (0∶00–0∶14 h:min) and active time 5∶38±1∶11 h:min (3∶50–7∶45 h:min) per day. However, a main effect of TC (P<0.001) was observed for rest, sitting, and moderate activity as well as for standing and active time (P  = 0.027). Rest and sitting increased during days 5–8 compared to the first days 1–5. On the contrary, standing, moderate activity and active time decreased.

### Physical Performance

**Figure 2:**
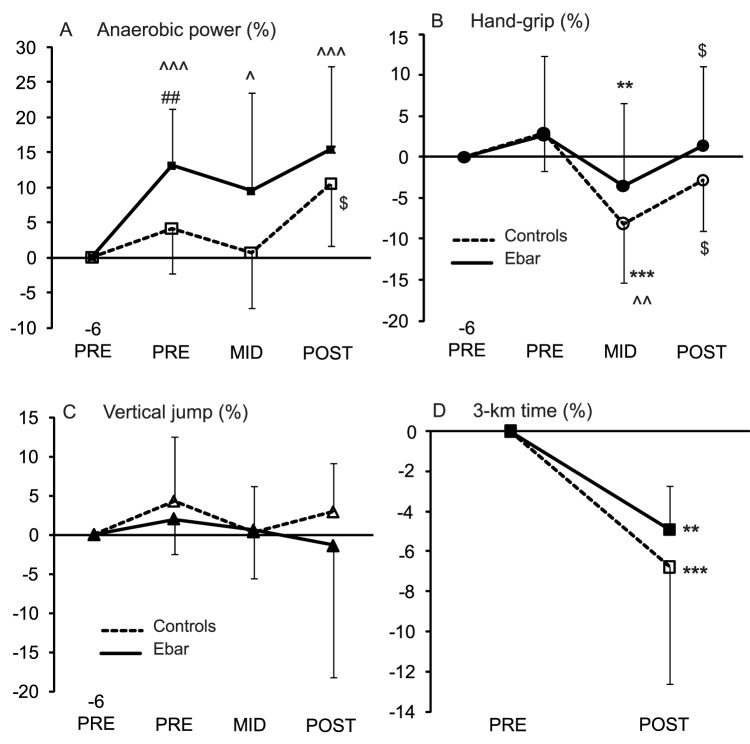
Changes (%) in physical performance in the control and Ebar groups during the study. Differences in change compared to the controls ## P<0.01. Differences in absolute change within the group compared to: -6 PRE ∧∧∧ P<0.001, ∧ P<0.05; PRE ***P<0.001, **P<0.01; MID $ P<0.05.

Relative changes in physical performance are presented in Figure 2. The following main effects were observed: the running time of 3 km, anaerobic power and hand-grip (P<0.001) of TC and the running time of 3-km (P  = 0.05) and anaerobic power (P  = 0.026) of the group. Hand grip decreased in both groups from PRE to MID but it recovered to the initial levels POST TC. The running time for 3 km improved in both groups as well. However, only the controls had an increase (P  = 0.022) in anaerobic power from the MID to POST. Maximum heart rate during the 3 km (Ebar PRE 191 bpm, POST 191 bpm; control PRE 186 bpm, POST 189 bpm), blood lactate after 3 km (Ebar PRE 11.5 mmol·L^−1^, POST 12.1 mmol·L^−1^; control PRE 10.6 mmol·L^−1^, POST 11.9 mmol·L^−1^), and vertical jump remained the same in both groups. While the Ebar group improved anaerobic power before the TC (6 day PRE to PRE, P<0.001), the relative change was higher than in the controls (P  = 0.007). Nevertheless, Ebar had higher anaerobic power than the controls in PRE (P  = 0.009), MID (P  = 0.046) and POST (P<0.028), there were no differences between the groups in its change during TC from PRE to POST.

### Questionnaire

**Figure 3:**
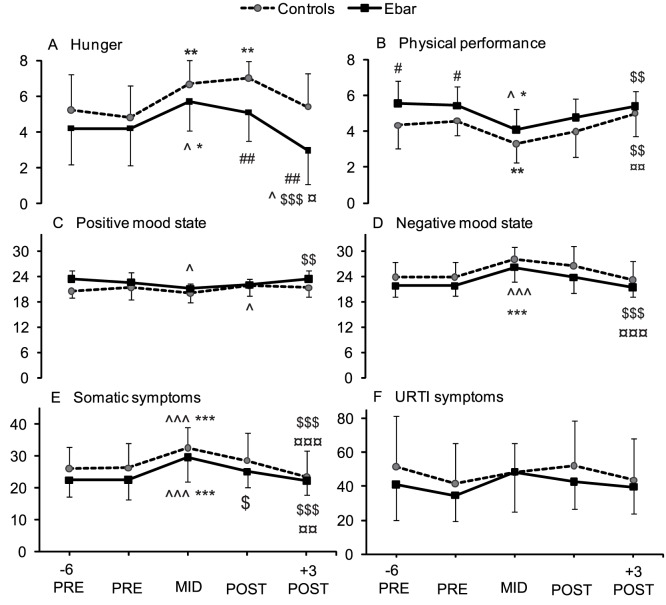
Questionnaire responses during the 8-day training course among the control and Ebar groups. Difference compared to: control ## P<0.01, # P<0.05; -6 PRE ∧∧∧ P<0.001, ∧ P<0.05; PRE ***P<0.001, **P<0.01, *P<0.05; MID $$$ P<0.001, $$ P<0.01, $ P<0.05; POST ¤¤¤ P<0.001, ¤¤ P<0.01, ¤ P<0.05.

Questionnaire data analysis (Figure 3) revealed significant main effects for both the TC and group for hunger (P<0.001; P  = 0.016, respectively), physical performance (P<0.001; P  = 0.009) and positive mood state (P  = 0.002; P  = 0.014). The Ebar group felt less hungry than the controls POST and +3d POST (P<0.01). Furthermore, Ebar felt themselves less hungry +3d POST compared to the MID (P<0.001), while the controls did not. In addition, Ebar evaluated that their physical performance was better compared to the controls -6d PRE and PRE (P<0.05), and they had an improved positive mood state from MID to +3d POST, while the controls did not. Negative mood state and somatic symptoms had a main effect of TC (P<0.001) by increasing from -6d PRE and PRE to MID and decreasing +3d POST to the PRE levels. Symptoms of URTI did not change.

#### Associations with physical activity, energy availability, water balanced and physical performance

While there were no differences between the groups in physical activity, energy availability and water balance, the groups were pooled for correlation analysis. Correlations between physical activity and energy availability, water balance, hand-grip and anaerobic power are presented in Table 4. Energy availability associated only negatively with sitting during the entire TC. Instead, water balance associated positively with sitting and negatively with moderate activity and active time. Change in hand-grip from PRE to MID correlated positively with sitting and negatively with moderate and active time on days 1–5, while change in anaerobic power from PRE to the MID correlated negatively with vigorous activity. Changes in performance tests did not associate with energy availability and water balance. Either change in BM was not related to change in the 3 km running time, but correlated negatively with change in anaerobic power from MID to POST (r  =  −0.46, P  = 0.021). However, negative mood state before TC associated negatively with energy availability during TC and sleeping time during the first half of TC (Table 5). In addition, negative mood state in the middle of the TC correlated negatively with active time and change in anaerobic power from PRE to MID (r  =  −0.40, P  = 0.048). Furthermore, negative association was observed between energy availability and somatic symptoms and URTI symptoms and between URTI symptoms and moderate activity and active time (Table 5).

**Table 4:** Pearson correlation coefficient between physical activity and energy availability, water balance and changes (Δ) in hand grip and anaerobic power during the 8-day training course.

		Energy availability	Water deficit	Hand gripΔPRE-MID	Anaerobic jumpΔPRE-MID
Sitting (h:min)	Days 1–5	−0.33	0.49*	0.40*	−0.09
	Days 5–8	−0.35	0.47*	−0.04	−0.09
	Days 1–8	−0.44*	0.53**	0.27	−0.07
Standing (h:min)	Days 1–5	0.10	0.46*	0.14	0.30
Moderate activity (h:min)	Days 1–5	0.05	−0.63***	−0.45*	−0.04
	Days 5–8	0.21	−0.59**	−0.16	−0.11
	Days 1–8	0.14	−0.64***	−0.37	−0.05
Vigorous activity (h:min)	Days 1–5	−0.11	−0.10	0.25	−0.42*
	Days 1–8	**−**0.20	**−**0.27	0.13	−0.40*
Active time (h:min)	Days 1–5	0.04	−0.63***	0.43*	**−**0.07
	Days 5–8	0.21	−0.58**	**−**0.18	**−**0.09
	Days 1–8	0.13	−0.64***	**−**0.36	**−**0.06

**Table 5:** Pearson correlation coefficient between physical activity, energy availability and water balance and questionnaire data during the 8-day training course.

		Negative mood state			Somatic symptoms			URTI symptoms				
		-6PRE	PRE	MID	-6PRE	PRE	MID	-6PRE	PRE	MID	POST	+3POST
Energy availability		* **−0.41**	* **−0.43**	**−**0.26	* **−0.40**	** **−0.50**	* **−0.45**	**−**0.25	* **−0.40**	**−**0.36	* **−0.49**	* **−0.47**
Water balance		0.06	0.12	0.36	0.37	0.35	* **0.41**	* **0.44**	** **0.51**	*** **0.60**	* **0.42**	0.27
Sleeping (h:min)	Days 1–5	**−**0.24	** **−0.47**	−0.28	−0.15	−0.23	−0.15	0.22	0.06	0.09	−0.23	−0.16
	Days 5–8	−0.07	−0.22	0.07	0.14	0.18	0.11	* **0.41**	0.35	* **0.47**	0.20	0.23
	Days 1–8	−0.17	*−**0.40**	−0.10	0.01	−0.01	−0.01	* **0.39**	0.26	0.34	0.00	0.06
Moderate activity (h:min)	Days 1–5	−0.08	−0.03	−0.34	−0.12	−0.15	−0.25	−0.24	−0.31	*−**0.48**	−0.34	−0.26
	Days 5–8	−0.18	−0.06	**−**0.51**	−0.06	−0.10	−0.20	−0.40	−0.29	* **−0.39**	**−**0.15	**−**0.11
	Days 1–8	**−**0.13	**−**0.05	* **−0.43**	**−**0.11	**−**0.13	**−**0.27	**−**0.32	**−**0.33	* **−0.48**	−0.30	−0.21
Active time (h:min)	Days 1–5	−0.06	−0.03	−0.34	−0.11	−0.14	−0.23	−0.23	−0.30	* **−0.48**	**−**0.33	**−**0.25
	Days 5–8	−0.19	−0.07	**−**0.51**	−0.06	−0.11	−0.22	*−0.40	−0.30	−0.38	−0.14	−0.09
	Days 1–8	−0.12	−0.05	*−**0.43**	−0.10	−0.13	−0.26	−0.32	−0.32	*−**0.47**	−0.28	−0.20

*** P<0.001,

** P<0.01,

* P<0.05.

## Discussion

The present study showed that additional easy-to-use protein-rich energy bars increased total energy intake and energy availability during a demanding wintertime military training course. Despite a higher energy supply, energy intake was insufficient in the Ebar group similarly to the control group. The participants in both groups consumed, however, only 57±13% of the provided energy during TC. The difference of this study to the previously published energy supplements studies during short-term military field training [12], [14], [15], [24]–[26] is the use of protein-rich supplement. However, like the previous studies, also in this study the use of supplements increased energy intake, although it could not prevent from energy deficit. Even energy availability was higher in the present Ebar group, it did not increase physical activity compared to the controls. Furthermore, an increase in aerobic physical performance after TC was observed in both groups. The reason that the additional 4.1 MJ•day^−1^ could not maintain energy balance might be that Ebar also had higher energy expenditure, although the difference between the groups was not significant. Energy expenditure was calculated by a method of intake/balance, which also takes into account the increased thermic effect of food of a higher protein intake and the carried combat gear of from 60 to 70 kg. Energy expenditure of physical activity was also increased by overall stress of TC [40].

In this study, increased somatic and URTI symptoms and negative mood state already before TC was related to lower energy availability during the training, which may indicate the lack of motivation for eating. In addition, increased somatic symptoms in the middle of TC were related to lower energy availability. Although Lieberman et al. [41] have found no effect of short 2-day caloric deprivation on mood state in laboratory environment, the result of insufficient energy intake despite sufficient provided food is in line with other military training studies. In a study of an 11-day cold-weather field exercise, the mean energy intake was 13.1 MJ·d^−1^ (3132 kcal·d^−1^), which was lower than rations offered from 17.6 to 21.8 MJ·d^−1^ (4200 to 5200 kcal·d^−1^) [8]. During the 28 day military training in hilly terrain, the average intake of participants with a ready-to-eat meal was 12.4 MJ·d^−1^ (2960 kcal·d^−1^) but they did not consume all of the 15.1 MJ·d^−1^ (3600 kcal·d^−1^) that they had [7]. However, the participants with lightweight rations consumed nearly all and the average intake of them during the training was 8.1 MJ·d^−1^ (1930 kcal·d^−1^). Both of these groups lost weight (4.3 kg vs. 1.1 kg, respectively) [7]. Popper et al. [9] found that eating less than usual is more obvious on the first day of the first combat situation, while the amount of eating increased on the second combat situation, but it was still less than usual. The three most likely reasons for this behavior in respective situations has been reported to be lack of time to prepare and eat food as well as not being hungry [9]. The inadequate energy intake in this study could be also a consequence of their previous experiences that an 8-day energy deficit could be tolerated because participants knew they would have a rest period after TC.

In the present study, association between physical activity and energy availability was not observed. This is in line with other studies among soldiers, where all subjects were expected to have a substantial energy deficit [6], [7] but not with studies where energy deficit was an option or self-selected [11], [13]. Similarly in a study by Mettler et al. [22], athletes were able to maintain their training volume and intensity during the 2-week period, despite an energy restriction of 60% of habitual energy intake and reduced well-being. Although enough energy was provided in this study the participants did not utilize it, which resulted in an energy deficit. Thus, the influence of energy intake on physical activity is not known exactly. However, in our study the more was negative mood state and URTI symptoms in the middle of TC, the less was physical activity indicating the role of motivation to perform tasks and be active. Even though the tasks were ordered to do as a group, often soldiers help each others in various tasks. In addition, by helping other group members in their duties, soldiers have possibility to make several behavioral choices, consciously or not, which may clearly affect their energy expenditure in spite of the same whole-day tasks assigned to them. This is supported by a wide variation in total physical activity time from 3∶50 to 7∶45 h:min per day.

While the energy expenditure is extremely high, 1.8 g·kg^−1^ intake of protein has been recommended [23]. In the present study, protein intake among Ebar was 2.1 g·kg^−1^. An easy-to-use protein-rich energy bar was thought to increase energy intake and thus decrease loss of FFM during strenuous TC. Ebar had a higher energy intake and energy availability compared to the controls. However, there were no differences between the groups in changes in FFM, energy deficit and physical activity, even Ebar had a trend for an increase in FFM, rather than a decrease. Among firefighters, eat-on-move snacks increased not only energy intake but also physical activity during a 2-d experimental period compared to ready-to-eat meal days [12]. Friedl et al. [42] have also shown the positive effects of using food supplementation during US Ranger training; 400 kcal•day^−1^ higher energy intake attenuated decrease FMM in energy deficit state in the presence of sleep deprivation, psychological stress and high physical activity. Previously, a protein intake of 1.8 g·kg^−1^ has been found to be ineffective in preventing a decline in FFM during the energy deficit of 4.2 MJ caused by increased energy expenditure [23]. In the present study, protein intake was sufficient to attenuate the decline in FFM during the exhaustive training, even though the energy deficit was 40%. This is in line with a study of recreational athletes who trained an average of 6 hours per week. Their protein intake was sufficient (∼2.3 g·kg^−1^) to maintain the lean body mass during the 40% energy restriction compared to the control group (∼1.0 g·kg^−1^) protein intake [22].

Increased protein intake has been found to produce satiation [43], increase energy expenditure by thermic effect of food, and decreased ad libitum energy intake [43] among obese, which are favorable phenomena during weight loss and maintenance. However, these phenomena could be unfavorable during a situation where exercise-induced energy expenditure is high and high-energy intake is recommended. In the present study, high-protein bars might have induced satiation in the Ebar group, leading to their inadequate energy intake from field rations, despite energy being sufficiently provided. This interpretation is supported by the question of hunger. Namely, the Ebar group felt themselves less hungry after TC than the controls. On the other hand, even while energy intake matched energy expenditure, an increased protein intake of 3.0 g·kg^−1^ has been shown to have a beneficial role during recovery after exercise by attenuating impairments in endurance performance [44]. The likely mediator of this potentially beneficial effect of protein feeding is perturbations in psychological symptoms of stress [44]. On the contrary, during weight loss among recreational athletes, no differences were observed between the low and high protein intake groups in satiety and total mood disturbance [22]. In the present study, even though there were no differences in physical performance between the Ebar and control groups, the questionnaire data revealed that Ebar group felt their physical performance and positive mood state better than the controls.

It would be assumed that the high activity level over 5 hours per day with only 4 hours rest would have induced decrements in physical performance. An improvement in the 3 km running time in both groups could be partly due to weight loss [45], but that was not the case. Possible adaptation to the testing procedure and an enhanced level of motivation and effort on the last day of the study [46] cannot be ruled out and may explain partly an increase in the 3 km running time. Improvement has also been observed in a study of Hodgon et al [47] after nine-day field training in cold winter weather: the time of the snowshoe course improved without any change in the anaerobic power test. During TC, participants carried combat gear weighing 60–70 kg, but the load carried in the 3 km test was much lighter, a 20 kg backpack. The subjects felt that this lighter load was much easier to carry during the 3 km test and they felt that they were “flying” during the running. Improved 3 km performance might also be a consensus of 8-day load carrying training, which decreased the energy cost of load-carrying [48]. Furthermore, the present findings confirm that lower body anaerobic power and vertical jump do not appear to be adversely affected by prolonged back load carriage [48]. The results also confirm the previous findings among athletes and military persons that the short-term (<10 days) consequences of underfeeding have a limited impact on muscle strength and aerobic and anaerobic endurance [22], [49], [50]. In contrast, also decreased lower body anaerobic power has been found after short period (72 h) of sustained operations [46] and after eight-day military field training [35]. In these studies, the time from a completion of the training course to the beginning of post testing was less than three hours. In the present study, the post test was performed 1 day after the training course, which may explain the difference between the results of anaerobic performance. Also loss of body mass was related to improvement in anaerobic power [51]. However, it has to take into account that longer period of underfeeding should definitely induced decrements in physical performance [3]. Contrary, hand-grip strength decreased in the middle of the training course in both groups and returned to the baseline levels one day after. Thus hand-grip strength might indicate an acute overall physiological and mental fatigue, even though it has not been found as a sensitive indicator of muscle mass loss and energy deficit [52].

Also the present results are in line with finding that low (∼1.0 g·kg^−1^) or high (∼2.3 g·kg^−1^) protein intake has no influence on changes in performance tests after hypoenergetic weight loss [22]. A daily carbohydrate intake of 5–6 g·kg^−1^ was lower than recommended for endurance athletes [53], but it was still enough to maintain anaerobic performance and even improve aerobic performance. For energy restricted strength and power athletes, above the 3.0 g•kg^−1^ carbohydrate intake has been recommended to maintain anaerobic performance [49]. Furthermore, improvement in 3 km running time could be a consequence of recently published evidence that after low carbohydrate intake during training period aerobic performance is improved after carbohydrate loading before the test [54]. In this study participants were able to rest 1 day after the training course before post test and load their energy stores.

The recommendation for fluid intake during military training in cold weather is 2 liters per day [55]. In both groups of the present study, ad libitum fluid intake was 2.2±0.5 L per day without water deficit. This confirms that the recommendations of 2 L per day is enough at group level, but is not enough for the most active participants while the activity was related to higher water deficit [56]. Even the variation in fluid intake (from 0.9 L·d^−1^ to 2.8 L·d^−1^) and water balance (from −1.7 L·d^−1^ to 0.8 L·d^−1^) was wide, water balance did not correlate with physical performance after the training course nor with change in FFM. In this study water balance was calculated by the differences between estimated water intake and water loss measured by deuterium elimination method. While 73% of the fat-free mass is water (Westerterp et al. 1995a), it could assume that the change in body mass loss is trough water deficit. However, in this study changes in TBW and FFM were nonsignificant and water deficit did not exist. Thus, there was no body mass loss through a water deficit.

### Conclusion

Based on the present study, an easy-to-use energy bar supplement of 4 MJ•day^−1^ did not prevent energy deficit or influence on PA during an 8-day strenuous military TC. A satiation effect of the high content of protein in the bars might have induced a decreased energy intake from field ration, since the Ebar group felt themselves less hungry after TC than the controls. However, negative mood state and URTI symptoms affected energy intake already before the training course. Thus mood state and health status are important factors in preventing undereating. In addition, during strenuous TC, PA seems to be primarily affected by factors other than energy supplementation such as mood state. The outcome and also a limitation of this study was the tremendous undereating. For evaluation the effects of increased energy intake on PA and performance, we should have had a third group which was forced to eat all provided food. Secondly to avoid biases, the subjects should be absolutely unaware of the group at they were included during the whole intervention period. In future studies, a special attention should be paid for the taste and variability of the food as well to maintain positive mood state in order to enhance energy intake during prolonged training.
